# The *Plasmodium falciparum*-Specific Human Memory B Cell Compartment Expands Gradually with Repeated Malaria Infections

**DOI:** 10.1371/journal.ppat.1000912

**Published:** 2010-05-20

**Authors:** Greta E. Weiss, Boubacar Traore, Kassoum Kayentao, Aissata Ongoiba, Safiatou Doumbo, Didier Doumtabe, Younoussou Kone, Seydou Dia, Agnes Guindo, Abdramane Traore, Chiung-Yu Huang, Kazutoyo Miura, Marko Mircetic, Shanping Li, Amy Baughman, David L. Narum, Louis H. Miller, Ogobara K. Doumbo, Susan K. Pierce, Peter D. Crompton

**Affiliations:** 1 Laboratory of Immunogenetics, National Institute of Allergy and Infectious Diseases, National Institutes of Health, Bethesda, Maryland, United States of America; 2 Malaria Research and Training Center, Faculty of Medicine, Pharmacy and Dentistry, University of Bamako, Bamako, Mali; 3 Biostatistics Research Branch, National Institute of Allergy and Infectious Diseases, National Institutes of Health, Bethesda, Maryland, United States of America; 4 Malaria Vaccine Development Branch, National Institute of Allergy and Infectious Diseases, National Institutes of Health, Bethesda, Maryland, United States of America; National Institute for Medical Research, United Kingdom

## Abstract

Immunity to *Plasmodium falciparum* (*Pf*) malaria is only acquired after years of repeated infections and wanes rapidly without ongoing parasite exposure. Antibodies are central to malaria immunity, yet little is known about the B-cell biology that underlies the inefficient acquisition of *Pf*-specific humoral immunity. This year-long prospective study in Mali of 185 individuals aged 2 to 25 years shows that *Pf*-specific memory B-cells and antibodies are acquired gradually in a stepwise fashion over years of repeated *Pf* exposure. Both *Pf*-specific memory B cells and antibody titers increased after acute malaria and then, after six months of decreased *Pf* exposure, contracted to a point slightly higher than pre-infection levels. This inefficient, stepwise expansion of both the *Pf*-specific memory B-cell and long-lived antibody compartments depends on *Pf* exposure rather than age, based on the comparator response to tetanus vaccination that was efficient and stable. These observations lend new insights into the cellular basis of the delayed acquisition of malaria immunity.

## Author Summary

*Plasmodium falciparum (Pf)* is a mosquito-borne parasite that causes over 500 million cases of malaria annually, one million of which result in death, primarily among African children. The development of an effective malaria vaccine would be a critical step toward the control and eventual elimination of this disease. To date, most licensed vaccines are for pathogens that induce long-lived protective antibodies after a single infection. In contrast, immunity to malaria is only acquired after repeated infections. Antibodies play a key role in protection from malaria, yet several studies indicate that antibodies against some *Pf* proteins are generated inefficiently and lost rapidly. The cells that are responsible for the maintenance of antibodies over the human lifespan are memory B-cells and long-lived plasma cells. To determine how these cells are generated and maintained in response to *Pf* infection, we conducted a year-long study in an area of Mali that experiences a six-month malaria season. We found memory B-cells and long-lived antibodies specific for the parasite were generated in a gradual, step-wise fashion over years despite intense *Pf* exposure. This contrasts sharply with the efficient response to tetanus vaccination in the same population. This study lends new insights into the delayed acquisition of malaria immunity. Future studies of the cellular and molecular basis of these observations could open the door to strategies for the development of a highly effective malaria vaccine.

## Introduction

To date, most successful vaccines have targeted pathogens that induce long-lived protective antibodies after a single infection, such as the viruses that cause smallpox, measles and yellow fever [1]. It has proved more difficult to develop highly effective vaccines against pathogens that do not induce sterile immunity such as the human immunodeficiency virus type-1 (HIV-1), *Mycobacterium tuberculosis* (Mtb), and *Plasmodium falciparum* malaria [2]. However, unlike HIV-1 and Mtb, clinical immunity to malaria can be acquired, but only after years of repeated *Pf* infections [3]. Passive transfer studies indicate that antibodies ultimately play a key role in protection from malaria [4], yet several studies show that antibodies to *Pf* antigens are inefficiently generated and rapidly lost in the absence of ongoing exposure to the parasite (reviewed in [5]). Elucidating the cellular basis of the inefficient acquisition of malaria immunity may ultimately prove critical to the design of an effective malaria vaccine.

Despite the key role that antibodies play in protection from a variety of infectious diseases, remarkably little is known about the cellular basis of acquiring humoral immunity in response to natural infections in humans. This gap in our knowledge is due in large part to the difficulty in studying natural infections in humans when we cannot predict who within a population will be infected with a given pathogen at a given time. Thus, our current understanding of the acquisition of immunity is largely derived from animal models and studies of humans after vaccination. These studies have established that long-lived, antibody-based immunity requires the generation and maintenance of memory B cells (MBCs) and long-lived plasma cells (LLPCs) (reviewed in [6], [7]). This process begins when naïve B cells bind antigen near the interface of B and T cell areas of secondary lymphoid organs. Several studies suggest that high-affinity binding drives naïve B cells to differentiate into short-lived, isotyped switched plasma cells (PCs) within the extra-follicular region which contributes to the initial control of infection. In contrast, lower affinity binding selects for entry of naïve B cells into follicles where germinal centers are formed. After a period of 7–10 days, through the CD4^+^ T-cell dependent process of somatic hypermutation, the germinal center reaction yields MBCs and LLPCs of higher affinity than the initial wave of short-lived plasma cells (SLPCs). MBCs recirculate and mediate recall responses after re-exposure to their cognate antigen by rapidly expanding and differentiating into PCs, whereas LLPCs residing in the bone marrow constitutively secrete antibody and provide a critical first line of defense against re-infection.

The mechanisms by which antibody responses are maintained over the human life-span remains an open question. In one model, LLPCs survive indefinitely in the bone marrow and independently maintain steady-state antibody levels [8]. Alternative models predict that PCs are replenished by MBCs that proliferate and differentiate in response to persistent [9] or intermittent exposure to antigen, and/or through non-specific by-stander activation (e.g. cytokines or TLR ligands) [10]. Unlike PCs, which are terminally-differentiated, MBCs may be maintained through homeostatic proliferation [11], possibly through exposure to polyclonal stimuli [10]. To address fundamental questions related to the generation and maintenance of MBCs and Abs specific for *Pf* malaria in children in malaria endemic areas, we conducted a year-long prospective study in a rural village of Mali that experiences an intense, sharply-demarcated six-month malaria season annually. We determined whether *Pf* infection generates MBCs specific for *Pf* blood stage antigens, and if so, whether they accumulate with age and cumulative *Pf* exposure, and also whether their frequency correlates with protection from malaria. In addition, we determined whether acute, symptomatic *Pf* infection resulted in an increase in the number of *Pf*-specific MBCs and the levels of specific antibodies, and if so, whether this increase remained stable over a six-month period of markedly reduced *Pf* transmission. By taking advantage of the tetanus immunization schedule in Mali in which infants and women of child-bearing age are vaccinated, we compared the relative efficiencies of the acquisition of tetanus toxoid (TT)- and *Pf*-specific MBCs and Ab, and also tested three hypotheses: 1) that growth of the MBC compartment depends on immunological experience rather than age, 2) that *Pf* infection induces non-specific activation of bystander B cells [12], [13], and 3) that polyclonal activation during heterologous immune responses is a general mechanism for maintaining MBCs and LLPCs [10].

## Results

### Malaria immunity is acquired gradually despite intense exposure to the *Pf* parasite

**Figure 1:**
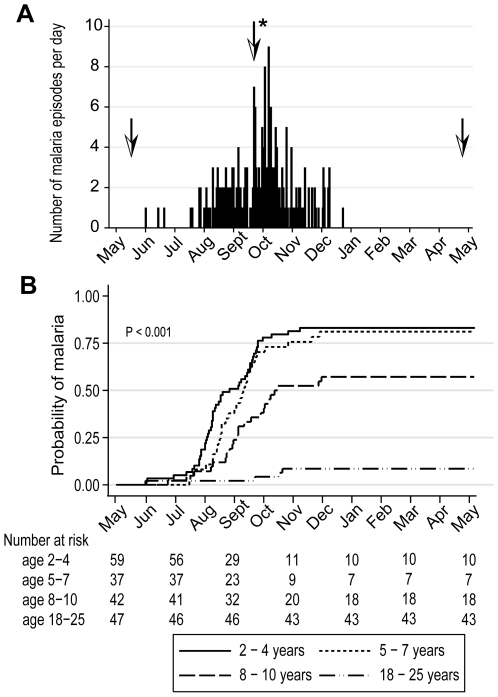
Malaria immunity is acquired gradually despite intense annual exposure to the *Pf* parasite. (A) Number of malaria episodes per day during the study period. Over a one-year surveillance period 185 individuals experienced 219 malaria episodes during a sharply-demarcated six-month malaria season. Malaria episodes were defined as fever ≥37.5°C and *Pf* asexual parasitemia ≥5000/µL blood. To track the B-cell response to acute malaria, and after a period of reduced *Pf* exposure, PBMCs and plasma were collected at points indicated by the arrows: before the malaria season, two weeks after the first malaria episode (arrow with asterisk indicates the mean time to first malaria episode, 132 days from enrollment), and six months after the end of the malaria season. (B) Kaplan-Meier estimates of the cumulative probability of malaria over the study period, according to age category. The number of individuals at risk over the study period is shown below the graph. The P value was obtained using the log rank test.

In May 2006 we initiated an observational cohort study in Mali to investigate the mechanisms underlying naturally-acquired malaria immunity. A detailed description of the study site and cohort has been reported elsewhere [14]. The study population was an age-stratified, random sample representing 15% of all individuals living in a small, rural, well-circumscribed, non-migratory community where antimalarial drugs were provided exclusively by the study investigators. During a two-week period one month prior to the abrupt onset of the six-month malaria season, we enrolled 225 individuals in four age groups: 2–4 years (n = 73), 5–7 years (n = 52), 8–10 years (n = 51), and 18–25 years (n = 49). Attendance at scheduled follow-up visits was >99% for children (2–10 years) and 82% for adults (18–25 years) during the one-year study period indicating a high degree of study awareness and participation. For the MBC analysis reported here, a subset of 185 individuals was randomly selected within each of the four age categories. All subsequent data and analysis refer to these 185 individuals. The baseline demographic and clinical characteristics of this subset are shown in Table 1, according to age group. As previously reported [14], only three of the characteristics shown in Table 1 were associated with decreased malaria risk in multivariate analysis—increasing age, sickle cell trait (HbAS), and asymptomatic *Pf* parasitemia at study enrollment. During the one-year study period there were 380 unscheduled clinic visits, during which 219 cases of malaria were diagnosed, five of which met the WHO criteria for severe malaria [15]. Malaria episodes were defined as an axillary temperature ≥37.5°C, *Pf* asexual parasitemia ≥5000 parasites/µL, and a non-focal physical exam by the study physician. As expected in this region of Mali, all malaria cases were confined to a six-month period that began in July, peaked in October, and ended by January (Fig. 1A). The incidence of malaria and the proportion of individuals experiencing at least one malaria episode decreased with age, whereas the time to the first malaria episode increased with age (Table 2 and Fig. 1B). Thus, despite intense annual *Pf* transmission at this study site, malaria immunity is acquired slowly.

**Table 1:** Baseline characteristics of the study cohort by age group.

	Age group, years				All (n = 185)
	2–4 (n = 59)	5–7 (n = 37)	8–10 (n = 42)	18–25 (n = 47)	
Gender, % female (no.)	66.1 (39)	48.7 (18)	33.3 (14)	61.7 (29)	54.1 (100)
Ethnicity, % (no.)					
Bambara	62.7 (37)	51.4 (19)	54.8 (23)	66.0 (31)	59.5 (110)
Sarakole	32.2 (19)	43.2 (16)	35.7 (15)	27.7 (13)	34.1 (63)
Fulani	3.4 (2)	5.4 (2)	7.1 (3)	4.3 (2)	4.9 (9)
Malinke	1.7 (1)	0.0 (0)	2.4 (1)	2.1 (1)	1.6 (3)
Hemoglobin AS, % (no.)a–	13.6 (8)	8.1 (3)	7.1 (3)	10.6 (5)	10.3 (19)
*P. falciparum* smear positive at enrollment, % (no.)b–	6.8 (4)	10.8 (4)	11.9 (5)	6.4 (3)	8.7 (16)
Parasitemia if smear positive at enrollment, parasites/microliter [geometric mean (95% CI)]	1438 (159–12973)	3616 (1500–8715)	415 (134–1287)	953 (39–23381)	1137 (579–2232)
GI helminth, % positive at enrollment (no.)c–	14.6 (8)	8.3 (3)	11.8 (4)	0 (0)	9.7 (15)
Urine schistosomiasis, % positive at enrollment (no.)d–	0 (0)	0 (0)	5.3 (2)	29.0 (9)	7.4 (11)
Distance lived from clinic, meters (mean ±SD)	395 (±116)	408 (±140)	365 (±83)	359 (±91)	382 (±110)
Bed net use, % (no.)e–	27.3 (15)	41.2 (14)	17.1 (7)	39.5 (15)	30.4 (51)

^a^–Data available for 177 subjects.

^b^–All subjects were asymptomatic at enrollment.

^c^–Data available for 154 subjects; GI = gastrointestinal.

^d^–Data available for 148 subjects.

^e^–Nightly bednet use self-reported at the end of the malaria season.

**Table 2:** Malaria clinical outcomes by age group.

	Age group, years				All (n = 185)
	2–4 (n = 59)	5–7 (n = 37)	8–10 (n = 42)	18–25 (n = 47)	
Malaria incidence, mean (±SD)a–	1.86 (±1.28)	1.81 (±1.17)	0.95 (±1.08)	0.09 (±0.28)	1.19 (±1.27)
Severe malaria incidence, no.b–	4	1	0	0	5
At least one malaria episode, % (no.)	83.1 (49)	81.1 (30)	57.1 (24)	8.5 (4)	57.8 (107)
Time to first malaria episode, days (median)c–	101	121	124	153	118
Parasitemia at first malaria episode, parasites/microliter [geometric mean (95% CI)]	39084 (30579–49954)	26417 (19440–35896)	20561 (15683–26956)	8816 (4082–19037)	28678 (24334 –33799)

^a^–Malaria episode defined as T≥37.5°C, asexual parasitemia ≥5000/microliter, and non-focal physical examination.

^b^–WHO definition of severe malaria.

^c^–Days since study enrollment.

### Analysis of *Pf*-specific and TT-specific MBCs and Abs in *Pf*-uninfected children and adults before the malaria season

We first established baseline levels of IgG^+^ AMA1-, MSP1- and TT-specific MBCs and Abs in *Pf*-uninfected, healthy children and adults in May just before the malaria season, a point at which there had been little to no *Pf* transmission for five months. For this analysis we excluded individuals with asymptomatic *Pf* parasitemia (8.7% of total cohort; Table 1), because they showed a decreased risk of malaria and tended toward higher frequencies of AMA1- and MSP1-specific MBCs and levels of Ab (data not shown).

**Figure 2:**
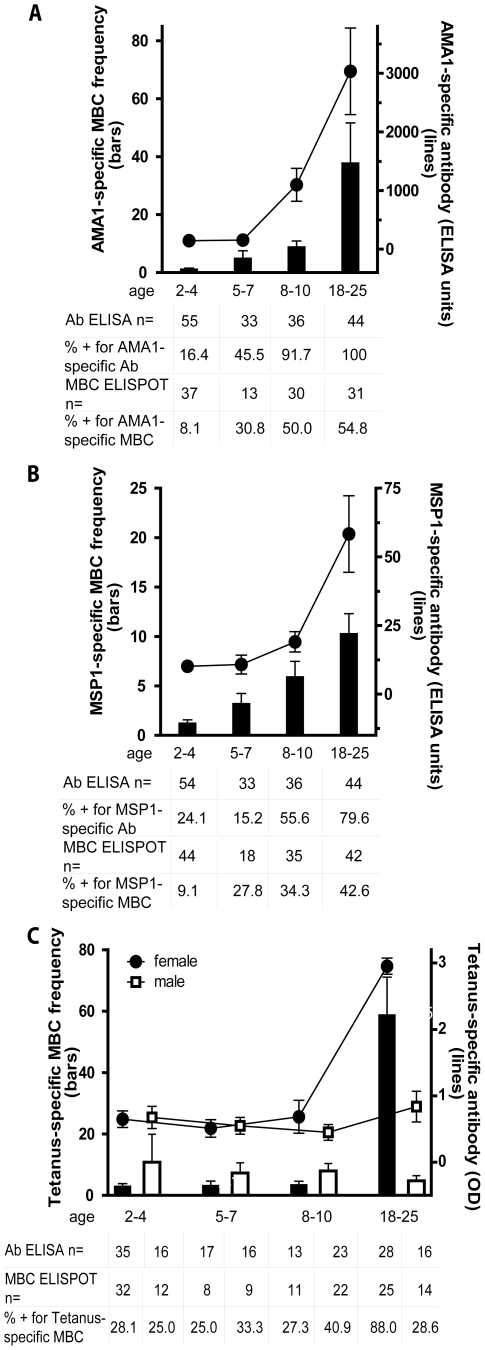
The *Pf*-specific MBC and long-lived antibody compartments expand gradually with age. Shown are the MBC frequencies (bars, left axis) and antibody levels (lines, right axis) specific for AMA1 (A) and MSP1 (B) by age category; and TT (C) by age category and gender; before the malaria season in *Pf*-uninfected individuals. The frequency of AMA1- and MSP1-specific MBCs increased with age (P<0.001 for both trends), as did the level of AMA1- and MSP1-specific antibodies (P<0.001 for both trends). There were no significant differences by gender for the AMA1- and MSP1-specific responses (not shown). To determine if the expansion of *Pf*-specific MBCs with age was driven by exposure to antigen or simply a function of age, we measured the TT-specific MBC and antibody response with age. In Mali, infants are vaccinated with TT, and females receive a TT booster around the age of 15 years to prevent neonatal tetanus. In contrast to AMA1 and MSP1, the frequency of TT-specific MBCs and the level of TT-specific antibodies for males did not change significantly from age 2 to 25 years (P = 0.80 and P = 0.44, respectively). However, the frequency of TT-specific MBCs and the level of TT-specific antibodies was higher in female adults compared to female children (P<0.001 for both comparisons). MBC frequencies were determined by ELISPOT and are expressed per million PBMC. The number of individual samples assayed and the percent of individual samples that exceeded the limit of detection (i.e. those considered positive) is indicated below the graph. The discrepancy in the sample size for ELISA data among 2–4 year olds is due to technical error during the performance of the ELISA. P values were obtained by the Kruskal-Wallis test. Data are shown as mean ± s.e.m.

We focused our analyses on MBCs and Abs specific for *Pf* blood-stage antigens because humoral responses are known to be critical to blood-stage immunity [4]. We examined the response to two blood stage proteins, Apical Membrane Antigen 1 (AMA1) and Merozoite Surface Protein 1 (MSP1), because we had previously studied the MBC and Ab responses to these antigens in vaccine trials of *Pf*-naïve individuals [16]. This afforded the opportunity to compare the acquisition of B cell memory to the same antigens after vaccination versus natural *Pf* infection. We express MBC data as ‘MBCs per 10^6^ PBMCs’, where ‘MBCs’ refers to the number of antibody secreting cells derived from MBCs during the six-day culture, and ‘10^6^ PBMCs’ refers to the number of PBMCs after culture. In the present study, the mean frequency of AMA1-specific MBCs per 10^6^ PBMCs increased with age (Fig. 2A; 2–4 yr: 1.2 [95% CI: 0.45–1.9]; 5–7 yr: 5.0 [95% CI: −0.2–10.1]; 8–10 yr: 8.9 [95% CI: 4.9–12.9]; 18–25 yr: 37.8 [95% CI: 10.4–65.3]; P<0.001), as did the proportion of individuals with detectable AMA1-specific MBCs (2–4 yr: 8.1%; 5–7 yr: 30.8%; 8–10 yr: 50.0%; 18–25 yr: 54.8%; P<0.001). Similarly, AMA1-specific Ab levels and the proportion of individuals seropositive for AMA1-specific Abs increased with age (Fig. 2A; P<0.001 for both comparisons). There was a positive correlation between the frequency of AMA1-specific MBCs and Ab levels (Spearman's correlation coefficient = 0.35; P = 0.005; Fig. S1).

We observed a similar age-associated increase in the frequency of MSP1-specific MBCs, although the overall frequency was lower than that for AMA1-specific MBCs (Fig. 2B; 2–4 yr: 1.2 [95% CI: 0.55–1.9]; 5–7 yr: 3.2 [95% CI: 1.2–5.2]; 8–10 yr: 5.9 [95% CI: 2.9–9.0]; 18–25 yr: 10.3 [95% CI: 6.3–14.3]; P<0.001). Likewise, the proportion of individuals who had detectable MSP1-specific MBCs (2–4 yr: 9.1%; 5–7 yr: 27.8%; 8–10 yr: 34.3%; 18–25 yr: 47.6%; P = 0.001) was similar to that for AMA1. MSP1-specific Ab levels and the proportion of individuals seropositive for MSP1-specific Abs also increased gradually with age (Fig. 2B; P<0.001 for both comparisons). There was a positive correlation between the frequency of MSP1-specific MBCs and Ab levels (Spearman's correlation coefficient = 0.34; P = 0.004; Fig. S1). Remarkably, despite exposure to 50–60 infective mosquito bites per month at the peak of each malaria season in this area [17], only approximately half of adults had detectable MBCs specific for AMA1 and MSP1, even though most had detectable AMA1- and MSP1-specific antibodies. Of the 72 individuals without detectable AMA1-specific MBCs before the malaria season, 64 (88.9% [95% CI 79.3–95.1]) did not have detectable MSP1-specific MBCs, suggesting that failure to generate MBCs to one *Pf* antigen is associated with failure to generate MBCs to other *Pf* antigens.

**Figure 3:**
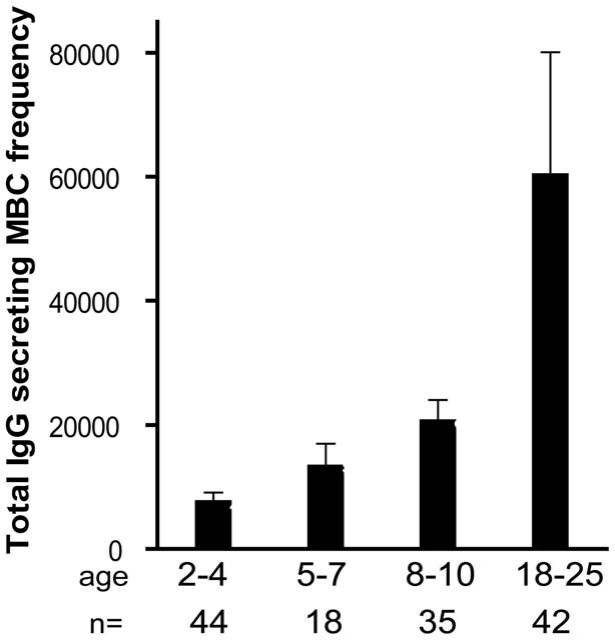
The size of total IgG^+^ MBC compartment expands gradually with age. The frequency of IgG^+^ MBCs per million PBMCs measured before the malaria season increased with age (P<0.001). The number of individuals in each age category is indicated. The *P* value was obtained by the Kruskal-Wallis test. Data are shown as mean ± s.e.m.

To understand if the expansion of *Pf*-specific MBCs with age was driven by repeated exposure to *Pf* antigens or simply a function of age, we determined the frequency of MBCs specific for an unrelated antigen, tetanus toxoid (TT), with age. In Mali, a single TT vaccine is administered to infants less than six months of age and a second TT vaccine is administered to females around 15 years of age to prevent neonatal tetanus. Thus, we measured TT-specific antibody and MBC responses at least 18 months after TT vaccination, a point at which the TT-specific response is likely to be at steady state. In contrast to what was observed for AMA1- and MSP1-specific MBCs, the frequency of TT-specific MBCs among males did not change significantly from age 2 to 25 years (Fig. 2C) (2–4 yrs: 10.8 [95% CI −7.4–29.0], 5–7 yrs: 7.3 [95% CI 0.7–13.9], 8–10 yrs: 8.0 [95% CI 3.1–12.8], 18–25 yrs: 4.7 [95% CI 1.4–8.1]; P = 0.80). Similarly, the proportion of male adults who were positive for TT-specific MBCs did not differ significantly from male children (2–4 yrs: 25.0%, 5–7 yrs: 33.3%, 8–10 yrs: 40.9%, 18–25 yrs 28.6%; P = 0.80). The slightly higher frequency of TT-specific MBCs in male versus female children was not statistically significant. However, the frequency of TT-specific MBCs was significantly higher in female adults compared to female children (Fig. 2C; mean frequency of TT-specific MBCs per million PBMC by age group (2–4 yrs: 2.9 [95% CI 1.1–4.7], 5–7 yrs: 3.2 [95% CI 0.2–6.1], 8–10 yrs: 3.4 [95% CI 1.1–5.7], 18–25 yrs: 58.7 [95% CI 34.2–83.3]; P<0.001) presumably the result of booster vaccination. Likewise, the proportion of female adults who were positive for TT-specific MBCs was significantly higher as compared to female children (2–4 yrs: 28.1%, 5–7 yrs: 25.0%, 8–10 yrs: 27.3%, 18–25 yrs 88.0%; P<0.001). For both females and males, TT-specific Ab levels mirrored MBC frequencies (Fig. 2C)—clearly increasing from female children to female adults (P<0.001), while not changing significantly by age in males (P = 0.44). Overall, TT-specific Ab levels and MBC frequencies correlated (Spearman's correlation coefficient = 0.48; P<0.001; Fig. S1). The observation that *Pf*-specific MBCs increased with age while TT-specific MBCs in individuals who received no booster vaccine did not increase and tended to decrease slightly with age indicates that the increase in *Pf*-specific MBCs is driven by repeated antigen exposure and is not simply a function of age. Of note, the size of the total IgG^+^ MBC compartment, as reflected in the peripheral blood, increased with age (Fig. 3; P<0.001), consistent with the maturation of the total MBC compartment with immunological experience.

### Longitudinal analysis of the *Pf*- and TT-specific MBC and Ab responses two weeks after acute malaria and after a prolonged period of decreased *Pf* exposure

**Figure 4:**
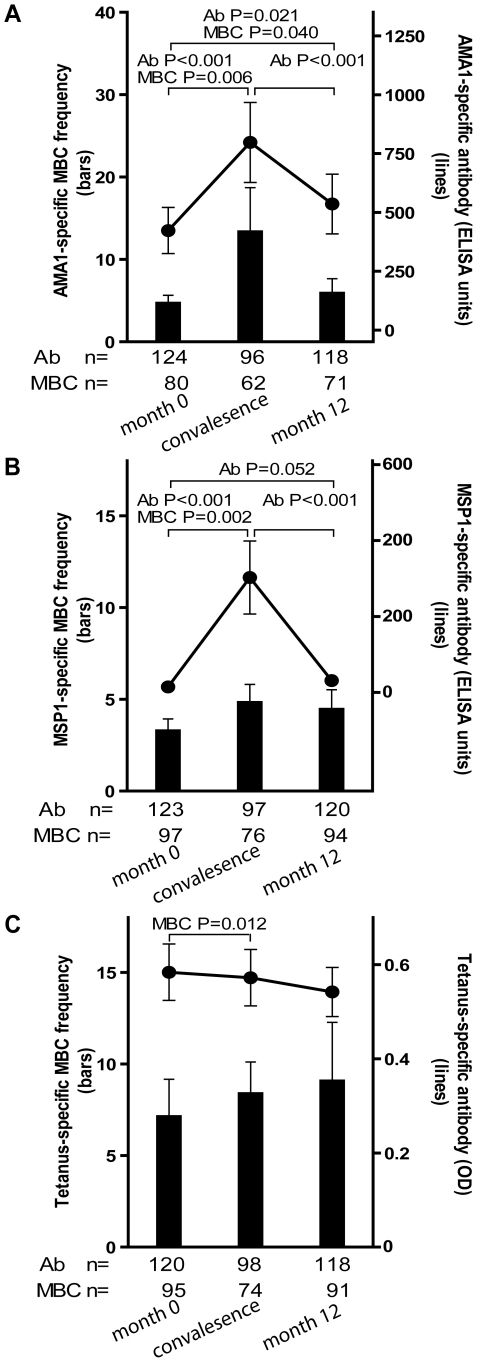
Longitudinal analysis of the *Pf*- and TT-specific MBC and antibody response. Compared to month zero, the MBC frequencies and antibody levels specific for AMA1 (A) and MSP1 (B) increased two weeks after the first episode of malaria and then contracted to a point slightly higher than pre-infection levels after a six-month period of decreased *Pf* exposure. Compared to month zero, there was a small but statistically significant increase in TT-specific MBC two weeks after the first episode of malaria (C), whereas the level of TT-specific antibodies did not change. The number of individuals in each age category is indicated. Only statistically significant P values are shown. P values were obtained by the Wilcoxon matched-pairs signed-rank test. Data are shown as mean ± s.e.m.

To assess the *Pf*-specific MBC and Ab responses to acute malaria, and to determine the stability of this response during a period of little to no *Pf* transmission, we measured the frequencies of MBCs and Ab levels specific for AMA1 and MSP1 14 days after the first episode of malaria (convalescence), and in a cross-sectional survey at the end of the following dry season (month 12), and compared these frequencies to the pre-malaria season baseline (month 0; as detailed above). Malaria episodes were defined as an axillary temperature ≥37.5°C, *Pf* asexual parasitemia ≥5000 parasites/µL, and a non-focal physical exam by the study physician. Because few adults experienced malaria (Table 2), this analysis only included children aged 2–10 years (see Fig. 4 for sample sizes at each time point). The mean frequency of AMA1-specific MBCs in children aged 2–10 years increased from month 0 to convalescence (Fig. 4A; month 0: 4.7 [95% CI: 2.8–6.6]; convalescence: 13.4 [95% CI: 2.7–24.1; P = 0.006] and then decreased from convalescence to month 12 (Fig. 4A; month 12: 5.9 [95% CI: 2.4–9.4]; P = 0.93 versus convalescence) to a point just above the frequency at month 0 (Fig. 4A; P = 0.021, month 0 vs. month 12). Likewise, the level of AMA1-specific Ab increased from month 0 to convalescence (Fig. 4A; month 0: 422.8 [95% CI: 228.7–617.0]; convalescence: 797.2 [95% CI: 460.0–1134.7; P<0.001], and then decreased from convalescence to month 12 (Fig. 4A; month 12: 535.5 [95% CI: 283.8–787.2]; P<0.001 versus convalescence], to a point just above month 0 levels (Fig. 4A; P = 0.040, month 0 vs. month 12).

The MSP1-specific MBC and Ab responses followed a similar pattern. The mean frequency of MSP1-specific MBCs in children aged 2–10 years increased from month 0 to convalescence (Fig. 4B; month 0: 3.3 [95% CI: 2.0–4.6]; convalescence: 4.8 [95% CI: 2.9–6.8; P = 0.002] and then decreased from convalescence to month 12 (Fig. 4B; month 12: 4.5 [95% CI: 2.4–6.6]; P = 0.71 versus convalescence) to a point just above the frequency at month 0 (Fig. 4B; P = 0.156, month 0 vs. month 12). Likewise, the level of MSP1-specific Ab increased from month 0 to convalescence (Fig. 4B; month 0: 14.6 [95% CI: 10.5–18.6]; convalescence: 302.6 [95% CI: 111.7–493.4; P<0.001], and then decreased from convalescence to month 12 (Fig. 4B; month 12: 31.1 [95% CI: 5.5–56.6]; P<0.001 versus convalescence], to a point just above month 0 levels (Fig. 4B; P = 0.052, month 0 vs. month 12).

To determine if malaria induces non-specific activation of ‘bystander’ MBCs, we compared the frequencies of TT-specific MBCs and Ab levels before the malaria season (month 0) to that 14 days after acute malaria (convalescence). We observed a small, but statistically significant increase in the frequency of TT-specific MBCs from month 0 to convalescence (Fig. 4C; month zero: 7.1 [95% CI: 3.1–11.2]; convalescence: 8.4 [95% CI: 5.0–11.8; P = 0.012) that did not change significantly at month 12 (month 12: 9.1 [95% CI: 3.2–15.4]; P = 0.974 versus convalescence]. In contrast, TT-specific Ab levels decreased slightly from month 0 to convalescence, and again from convalescence to month 12, although neither decline was statistically significant (Fig. 4C; month 0: 0.58 [95% CI: 0.5–0.7]; convalescence: 0.57 [95% CI: 0.5–0.7; P = 0.063]; month 12: 0.54 [95% CI: 0.4–0.6]; P = 0.525 versus convalescence). Collectively these results indicate that malaria infection results in an increase in the frequencies of both *Pf*-specific, and bystander MBCs. However, malaria selectively induces *Pf*-specific Ab production but does not appear to drive the differentiation of bystander naïve and memory B cells into PCs.

### B cell subsets in *Pf*-uninfected children and adults before the malaria season

**Figure 5:**
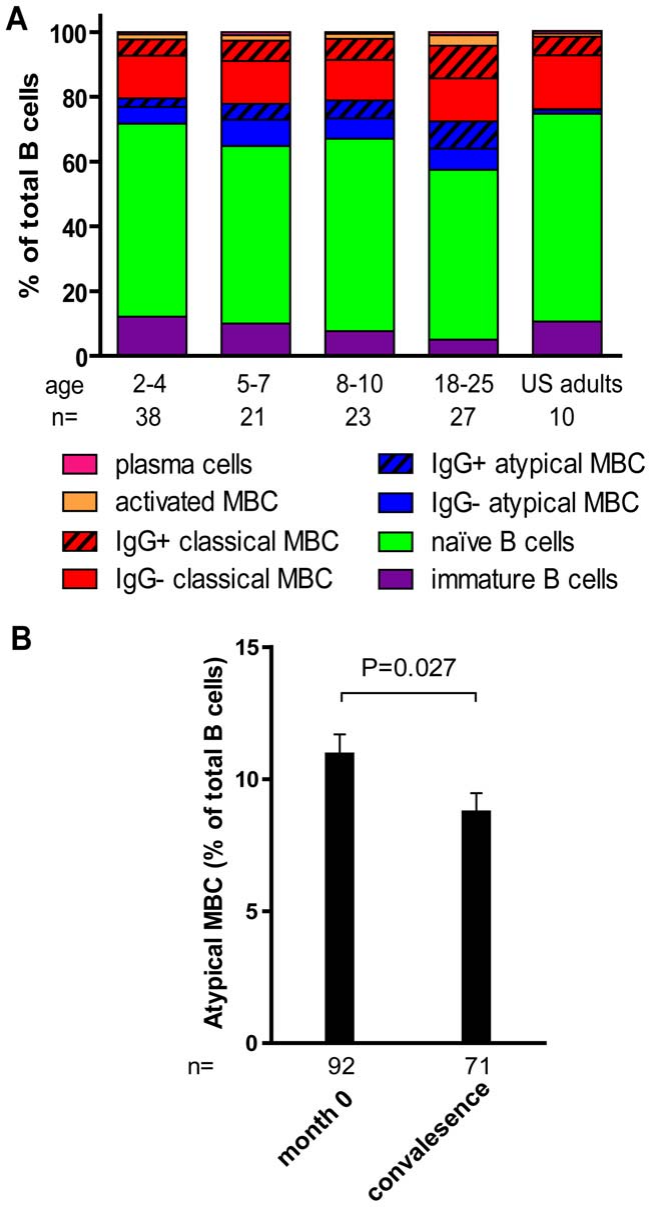
Profile of B-cell subsets before the malaria season in children and adults. (A) By flow cytometry the following B cell subsets were quantified from samples collected before the malaria season: immature B cells CD19^+^ CD10^+^, naïve B cells CD19^+^ CD27^−^ CD21^+^ CD10^−^, atypical MBCs CD19^+^ CD27^−^ CD21^−^ CD10^−^, classical MBCs CD19^+^ CD27^+^ CD21^+^, activated MBCs CD19^+^ CD21^−^ CD27^+^ CD20^+^, and PCs CD19^+^ CD21^−^ CD20^−^ CD10^−^. As a percentage of CD19^+^ B cells, immature B cells (P<0.001) and naïve B cells (P = 0.047) decreased with age, while atypical MBCs (P = 0.002), IgG^+^ atypical MBCs (P<0.001), IgG^+^ classical MBCs (P<0.001)), activated MBCs (P = 0.001), IgG^+^ activated MBCs (P<0.001) and PCs (P = 0.046) increased with age. P values were obtained by the Kruskal-Wallis test. (B) As a percentage of CD19^+^ B cells, atypical MBCs decreased 14 days after acute malaria in children aged 2–10 years compared to the percentage before the malaria season. The P value was obtained by the Wilcoxon matched-pairs signed-rank test. Data are shown as mean ± s.e.m.

By FACS we determined the proportion of B cell subsets in individuals (2–4 yrs [n = 38], 5–7 yrs [n = 21], 8–10 yrs [n = 23], 18–25 yrs [n = 27]) before the malaria season (Fig. 5A). With increasing age, and as a percentage of total CD19^+^ B cells we observed a decrease in immature B cells (CD19^+^ CD10^+^; P<0.001) and naïve B cells (CD19^+^ CD27^−^ CD21^+^ CD10^−^; P = 0.047) and an increase in resting IgG^+^ MBCs (CD19^+^ CD27^+^ CD21^+^; P<0.001) and activated IgG^+^ MBCs (CD19^+^ CD27^+^ CD21^−^ CD20^+^ CD10^−^; P<0.001). The increase with age of classical MBCs is consistent with the increase in total IgG^+^ MBCs we observed using the MBC ELISPOT assay (Fig 3).

In a subset of 87 individuals from this same study cohort, we previously reported that *Pf* exposure is associated with an expanded subset of ‘atypical’ MBCs that express FCRL4 and are hyporesponsive to *in vitro* stimuli [18], similar to the ‘exhausted’ MBCs described in viremic, HIV-infected individuals [19]. Atypical MBCs are defined as CD19^+^ CD27^−^ CD21^−^ CD20^+^ CD10^−^ and typically represents <4% of circulating CD19^+^ B cells in healthy U.S. adults [19]. Here, analyzing a larger number of individuals in the cohort, we confirmed that this subset of MBCs is expanded in Malian children and adults compared to malaria-naïve U.S. adults (U.S. adults: 1.4% [95% CI: 0.9–1.8]; Malian children aged 2–10 years: 10.2% [95% CI: 8.7–11.8], P<0.001 versus U.S. adults; Malian adults aged 18–25 years: 14.8% [95% CI: 11.0–19.1], P<0.001 versus U.S. adults). Thus, in addition to the increase in classical MBCs, an ‘atypical’ MBC subset is expanding with age in this study population.

### Longitudinal profiling of B cell subsets in children before and after acute malaria

We investigated the impact of acute malaria on the relative proportion of B cells in each subset in children aged 2–10 years. Compared to the pre-malaria season baseline (month 0), there were no significant changes in the percent of lymphocytes that were CD19^+^ 14 days after acute malaria. Within the CD19^+^ B cell population there were no significant changes in the percent of immature B cells, naïve B cells, or resting MBCs, after acute malaria. Moreover, there was no change in the proportions of resting and atypical MBCs that were IgG^+^. However, we observed a decrease in the percentage of total atypical MBCs (Fig. 5B; month 0: 10.9% [95% CI: 9.4–12.4], convalescence: 8.7% [95% CI: 7.3–10.2]; P = 0.027), and an increase in activated MBCs following acute malaria (month 0: 1.6 [95% CI: 1.2–2.0], convalescence: 1.9 [95% CI: 1.4–2.4]; P = 0.09). Within the activated MBC subset there was a significant increase in the proportion that were IgG^+^ (month 0: 59.0% [95% CI: 56.0–62.1], month 12: 62.8% [95% CI: 59.2–66.3]; P<0.001). The decrease in the proportion of atypical MBCs in the peripheral blood suggests that this subpopulation may be trafficking out of the circulation into tissues in response to acute malaria.

### AMA1- and MSP1-specific MBC frequencies and Ab levels and malaria risk

We determined prospectively whether AMA1- or MSP1-specific Ab levels or MBC frequencies measured just prior to the six month malaria season were associated with the subsequent risk of malaria. For this analysis a malaria episode was defined as an axillary temperature ≥37.5°C, *Pf* asexual parasitemia ≥5000 parasites/µL, and a non-focal exam by the study physician. Because the incidence of malaria was very low in adults during the study period (Table 2), they were excluded from this analysis. Three measures of malaria risk were analyzed: 1) whether or not malaria was experienced, 2) the incidence of malaria, and 3) the time to the first malaria episode. In the corresponding multivariate regression models (logistic, Poisson, and Cox regression) which controlled for age, sickle cell trait, and concurrent asymptomatic *Pf* parasitemia, we found no correlation between malaria risk and AMA1- or MSP1- specific Ab levels or MBC frequencies. As discussed below, this finding was not unexpected based on the observation that the malaria vaccine candidates AMA1 and MSP1 did not confer protection against malaria in clinical trials [20], [21].

## Discussion

In this year-long prospective study of children and adults in an area of intense, annual, sharply demarcated *Pf* transmission, we show that MBCs specific for *Pf* can be acquired, but only gradually in a stepwise fashion over years of repeated *Pf* exposure. MBCs specific for two *Pf* antigens, AMA1 and MSP1, increased in frequency in response to acute *Pf* infection, and then contracted during a six-month period of decreased *Pf* exposure to a point slightly above pre-infection levels. Cross-sectional analysis of individuals aged 2–25 years just before the malaria season indicated that this step-wise, incremental increase in *Pf*-specific MBCs with each malaria season contributes to the gradual expansion of the *Pf*-specific MBC compartment with cumulative *Pf* exposure. By comparison, the stable frequency of TT-specific MBCs with age after immunization in infancy indicates that growth of antigen-specific MBC compartments does not simply occur with age, but requires repeated antigen exposure. We do not formally know if the gradual gain in *Pf*-specific MBCs is in fact due to an increase in long-lived MBCs, or whether those MBCs require *Pf*-stimulation and would be lost if *Pf* transmission did not resume after the six-month dry season. In another setting, namely in an area of Thailand with low *Pf* transmission, Wipasa *et al.* [22] recently reported that nearly half of adults studied had acquired long-lived *Pf*-specific MBCs as a result of infrequent malaria infections. It will be of genuine interest to understand the cellular and molecular mechanisms at play in the generation of MBCs under these very different conditions of exposure of children versus adults as these could have significance with regard to vaccine development. Moreover, recent studies in mouse models are revealing multiple, phenotypically and functionally distinct populations of MBCs [23], [24] and it will be of interest to further characterize *Pf*-specific MBCs in different malaria endemic settings.

The study described here provides a rare view of the acquisition and maintenance of human B cell memory. Most prospective studies of human B and T cell immunological memory have evaluated responses to vaccination rather than natural infection, in part because of the difficulty of predicting who within a population will be infected with a given pathogen at a given time. In response to a single vaccination, several studies have described an expansion and contraction of vaccine-specific MBCs [25], [26] and CD8^+^ memory T cells [27]. In one of the few longitudinal studies of the MBC response to natural infection, Harris *et al*. examined antigen-specific MBC responses of patients presenting with acute *Vibrio cholerae* infection, a pathogen that elicits long-term protective immunity against subsequent disease [28]. In contrast to our results, they observed that the majority of patients acquired IgA and IgG MBCs specific for two *Vibrio cholerae* antigens and that these persisted up to one year after infection.

Whereas MBCs mediate recall responses to reinfection by rapidly expanding and differentiating into PCs, LLPCs residing in the bone marrow constitutively secrete antibody in the absence of antigen and thus provide a critical first line of defense against reinfection [6]. Logistical constraints precluded the direct measurement of circulating PCs in this study. However, we took advantage of the discrete six-month dry season, a period of little to no *Pf* transmission, to infer the relative contributions of SLPCs and LLPCs to the *Pf*-specific IgG response based on a serum IgG half-life of ∼21 days [29]. Two weeks after acute malaria, AMA1- and MSP1-specific Ab levels increased significantly and then decreased over a six-month period to a point just above pre-infection levels, indicating that the majority of PCs generated in response to acute *Pf* infection were short-lived. This observation is consistent with previous studies that described rapid declines in *Pf*-specific Ab within weeks of an acute malaria episode [30], [31]. We infer that the small net increase in *Pf*-specific Ab at the end of the six-month dry season represents the acquisition of *Pf*-specific LLPCs. Because *Pf* transmission resumes after the six-month dry season, we cannot estimate the long-term decay rate of *Pf*-specific Ab in the absence of reinfection. It remains to be seen whether long-term decay rates of *Pf*-specific Ab are comparable to rates of Ab decay after exposure to common viral and vaccine antigens such as mumps and measles, for example, which elicit Ab with half-lives exceeding 200 years [32]. The small incremental gains in AMA1- and MSP1-specific Abs in response to acute malaria mirrors the gradual exposure-related increase in *Pf*-specific MBCs, consistent with the long-lived Abs being the products of LLPCs derived from MBCs. Unlike the response to some other pathogens, such as measles, which induce long-lived protective Abs after a single exposure, it may be that repeated exposure to the *Pf* parasite is necessary to ‘fill’ the *Pf*-specific LLPC compartment to the point where basal levels of circulating Abs to any given *Pf* antigen reach a protective threshold. In a separate study of this cohort, we observed a similar pattern of transient increases during the malaria season of Abs specific for a large number of *Pf* antigens using protein microarrays [33] suggesting that malaria induces a relatively high SLPC-to-LLPC ratio that is not exclusively a function of the inherent qualities of any given antigen *per se*.

In contrast to the highly efficient immune response to a single smallpox vaccination, which generates long-lived (>50 years) MBCs in nearly all vaccinees [34], a remarkably high proportion of adults in the present study did not have detectable AMA1- or MSP1-specific MBCs despite annual exposure to 50–60 infective mosquito bites per person per month at the height of the malaria season [17], similar to what Dorfman *et al.* observed in a cross-sectional study in Kenya [35]. Importantly, most female adults in the present study had detectable TT-specific MBCs three to ten years after a single TT booster vaccine in adolescence. We previously reported that AMA1- and MSP1-specific MBCs were reliably generated in *Pf*-naïve U.S. adults following just two vaccinations [16]. Taken together, these observations indicate that the relatively inefficient generation and/or maintenance of *Pf*-specific MBCs in response to natural *Pf* infection cannot be ascribed entirely to inherent deficiencies in the antigens themselves. Collectively, these observations raise a central question: if AMA1 and MSP1-specific MBCs and Abs can be efficiently generated by vaccination of *Pf*-naïve adults, and TT-specific MBCs and Abs can be efficiently generated by vaccination of *Pf*-exposed individuals in this cohort, what underlies the inefficient acquisition and/or maintenance of AMA1 and MSP1-specific MBCs and Abs in response to natural *Pf* infection? One simple answer, in addition to parasite antigenic variation [36], [37], might be that the enormous number of antigens encoded by the over 5,400 *Pf* genes overwhelms the immune system's capacity to select for and commit a sufficient number of MBCs and LLPCs specific for any given *Pf* antigen to a long-lived pool [38]. If immunity to clinical malaria requires high levels of antibodies to a large number of *Pf* proteins, then the inability to commit large numbers of MBCs and LLPCs specific for any given *Pf* antigen during any given infection, as shown here, may explain, in part, why malaria immunity is acquired slowly. In this scenario the *Pf*-infected individual is capable of the normal generation and maintenance of MBCs and LLPCs, but acquiring a sufficient number of MBCs and LLPCs to a large number of antigens may simply take years.

It is also possible that *Pf* infection disrupts the immune system's ability to generate or maintain MBCs or LLPCs. The differentiation of B cells into long-lived MBCs depends to a great extent on the affinity of their BCRs for antigen. Recently, evidence was presented that affinity maturation of B cells may fail to occur in the absence of adequate Toll-like receptor (TLR) stimulation [39]. We recently reported that Malian adults were relatively refractory to CpG, a TLR9 agonist incorporated into two subunit malaria vaccine candidates [40], raising the possibility that the slow acquisition of MBCs observed here may be due to a failure of B cells to undergo affinity maturation during *Pf* infection. Although our data do not directly address the role of apoptosis in the gradual acquisition of *Pf*-specific MBCs, it is worth noting that we found no evidence of *Pf*-induced ablation of *Plasmodium*-specific MBCs, as was observed in mice four days after *Plasmodium yoelii* infection [41]. The relatively inefficient response to natural *Pf* infection also does not appear to be due to a persistent, *Pf*-induced general immunosuppression as the frequency of TT-specific MBCs increased significantly in most adult females in response to a single TT booster vaccination, an increase that appeared to be maintained for years. In an experimental model of lymphocytic choriomeningitis virus (LCMV) infection, a high antigen-to-B cell ratio disrupted germinal center formation and the establishment of B cell memory [42]. It is plausible that a similar mechanism is at play during the blood stage of *Pf* infection when the immune system encounters high concentrations of parasite proteins. Indeed, germinal center disruption is observed in mice infected with *P. berghei* ANKA [43] and *P. chabaudi* [44]. It is also possible that specific parasite products selectively interfere with the regulation of B cell differentiation [45] or with the signals required for sustaining LLPCs in the bone marrow [46]. It is also conceivable that the disproportionately high level of class-switched SLPCs we observed in response to *Pf* infection arises from pre-diversified IgM^+^IgD^+^CD27^+^ (marginal zone) B cells—analogous to the rapid protective response against highly virulent encapsulated bacteria that do not elicit classical T-dependent responses [47]. These and other hypotheses could be tested by applying systems biology methods [48] and targeted *ex vivo* and *in vitro* assays to rigorously conducted prospective studies of *Pf*-exposed populations.

We previously reported that *Pf* exposure is associated with a functionally and phenotypically distinct population of FCRL4^+^ hypo-responsive atypical MBCs [18], similar to the ‘exhausted’ MBCs described in HIV-infected individuals [19]. In this study, with a larger sample size, we confirmed that *Pf* exposure is associated with an expansion of FCRL4^+^ MBCs. The accumulation of atypical MBCs could be linked to the slow acquisition of *Pf*-specific MBCs, as naïve B cells in response to *Pf* infection could have a propensity to differentiate into atypical rather than classical MBCs. We also observed that the FCRL4^+^ MBC population decreased in the peripheral circulation two weeks after acute malaria suggesting that these MBCs are directly involved in the response to *Pf* infection, possibly trafficking to secondary lymphoid tissues. Although the function of FCRL4^+^ MBCs is not established, Moir *et al*. [19] suggested that FCRL4^+^ ‘exhausted MBCs’ contribute to the B cell deficiencies observed in HIV-infected individuals. In contrast, Ehrhardt *et al*.[49], who first described FCRL4^+^ ‘tissue-like MBCs’ in lymphoid tissues associated with epithelium, suggested that these cells may play a protective role during infections. At present, the factors that underlie the expansion of atypical MBCs in this study population are not known. Genetic or environmental factors that are associated with *Pf* transmission but not accounted for in this study could explain this observation. It will be of interest to understand the origin, antigen-specificity, and function of FCRL4^+^ MBCs in the context of *Pf* infection and the potential impact of these MBCs on the ability of children to respond to malaria vaccines.

In multivariate analysis we found no correlation between the frequency of MBCs and levels of Abs specific for AMA1 or MSP1 and malaria risk. This is not necessarily unexpected in light of recent clinical trials that showed that vaccination with either AMA1 or MSP1 did not confer protection [20], [21]. Furthermore, we suspect that the frequency of MBCs *per se* may not reliably predict clinical immunity to malaria regardless of antigen specificity. Malaria symptoms only occur during the blood stages of *Pf* infection and can begin as early as three days after the blood stage infection begins [50].Because the differentiation of MBCs into PCs peaks ∼6–8 days after re-exposure to antigen [10], there may not be sufficient time for MBCs specific for *Pf* blood stage antigens to differentiate into the antibody-secreting cells that would prevent the onset of malaria symptoms. In contrast, the longer incubation period of other pathogens allows MBCs to differentiate into protective antibody-secreting cells before symptoms develop. For example, follow-up studies of hepatitis B vaccinees have shown that protection can persist despite the decline of hepatitis B-specific antibodies to undetectable levels [51], presumably due to the recall response of persistent MBCs. Thus, protection against the blood stages of malaria may depend on achieving and maintaining a critical level of circulating antibody that can rapidly neutralize the parasite. MBCs may contribute to the gradual acquisition of protective immunity by differentiating into LLPCs with each *Pf* infection.

Here we also provide evidence concerning the mechanism by which MBCs and LLPCs are maintained. We observed a modest but statistically significant increase in TT-specific MBCs two weeks after acute malaria, in support of the hypothesis that MBCs are renewed by polyclonal or ‘bystander’ activation [10]. The stable frequency of TT-specific MBCs with age suggests that the small increases associated with *Pf*-induced polyclonal activation are matched by the rate of loss of senescent TT-specific MBCs. It has also been proposed that non-specific polyclonal stimulation maintains long-lived Ab responses by driving MBCs to differentiate into SLPCs or LLPCs [10]. Similarly, it has been hypothesized that *Plasmodium* infection generates large amounts of non-specific Ig [52] through polyclonal B cell activation [12], [13]. However, despite the presence of TT-specific MBCs and their expansion following *Pf* infection, we did not observe a concomitant increase in TT-specific IgG. This finding is consistent with recent human studies that demonstrate a lack of bystander IgG production after heterologous vaccination or viral infection [32], [53]; as well as studies in mice that demonstrate PC persistence after MBC depletion [54], and the failure of MBCs to differentiate into PCs *in vivo* upon TLR4 and 9 activation [55]. This finding does not represent an overt inability of TT-specific MBCs to differentiate into PCs, since adult females in this study had a sharp increase in tetanus IgG after a single tetanus booster. It is possible that bystander MBCs specific for antigens other than TT differentiate into PCs after *Pf* infection, but based on the results of this study we hypothesize that the preponderance of IgG produced in response to malaria is specific for the ∼2400 *Pf* proteins expressed during the blood-stage of infection [56], and that increases in ‘non-specific’ IgG reflect boosting of cross-reactive B cells [57], [58]. From a basic immunology perspective, these data support a model in which non-specific stimuli contribute to MBC self-renewal, but not to the maintenance of LLPCs. Studies of other Ab specificities and isotypes before and after malaria and other infections would test this hypothesis further. Although a recent mouse study showed that MBCs do not proliferate *in vivo* after immunization with an irrelevant antigen [59], this may reflect the difference in requirements for MBC maintenance in mammals with relatively short life spans.

It is of general interest to determine which parasite products are responsible for the polyclonal activation of MBCs observed here. Studies *in vitro* suggest that *Pf* drives polyclonal MBC activation by the cysteine-rich interdomain regions 1α (CIDR1α) of the *Pf* erythrocyte membrane protein 1 (PfEMP1) [13], [60], but it is conceivable that *Pf*-derived TLR agonists [61], [62] or bystander T cell help [63], [64], [65] also contribute to MBC proliferation in the absence of BCR triggering [66].

Animal models have provided important insights into the immunobiology of *Plasmodium* infection [67], but ultimately, despite obvious experimental limitations, it is critical to investigate the human immune response to *Pf* in longitudinal studies since findings from animal models do not always mirror human biology or pertain to the clinical context [68], [69]. Key challenges for future studies will be to determine the molecular basis of the inefficient generation of MBCs and LLPCs in response to *Pf* infection and to determine the longevity of these cells in the absence of *Pf* transmission over longer periods of time. Greater insight into the molecular and cellular basis of naturally-acquired malaria immunity could open the door to strategies that ultimately prove useful to the development of a highly effective malaria vaccine.

## Materials and Methods

### Ethics statement

The ethics committee of the Faculty of Medicine, Pharmacy, and Odonto-Stomatology, and the institutional review board at the National Institute of Allergy and Infectious Diseases, National Institutes of Health approved this study (NIAID protocol number 06-I-N147). Written, informed consent was obtained from adult participants and from the parents or guardians of participating children.

### Study site

This study was carried out in Kambila, a small (<1 km^2^) rural village with a population of 1500, located 20 km north of Bamako, the capital of Mali. *Pf* transmission is seasonal and intense at this site from July through December. The entomological inoculation rate measured in a nearby village was approximately 50–60 infective bites per person per month in October 2000 and fell to near zero during the dry season [17]. A detailed description of this site and the design of the cohort study has been published elsewhere [14].

### Sampling strategy, study participants, and malaria case definition

In May 2006, during a two-week period just prior to the malaria season, 225 individuals aged 2–10 years and 18–25 years were enrolled after random selection from an age-stratified census of the entire village population. Enrollment exclusion criteria were hemoglobin <7 g/dL, fever ≥37.5°C, acute systemic illness, use of anti-malarial or immunosuppressive medications in the past 30 days, or pregnancy. All analysis in the present study pertains to an age-stratified subset of individuals (n = 185) randomly selected from those who had complete sets of PBMC samples over the entire study period. From May 2006 through May 2007, participants were instructed to report symptoms of malaria at the village health center, staffed 24 hours per day by a study physician. For individuals with signs or symptoms of malaria, blood smears were examined for the presence of *Pf*. Patients with positive smear results (i.e. any level of parasitemia) were treated with a standard 3-day course of artesunate plus amodiaquine, following the guidelines of the Mali National Malaria Control Program. Anti-malarial drugs were provided exclusively by the study investigators. Children with severe malaria were referred to Kati District Hospital after an initial parenteral dose of quinine. For research purposes, a malaria episode was defined as an axillary temperature ≥37.5°C, *Pf* asexual parasitemia ≥5000 parasites/µL, and a nonfocal physical examination by the study physician. Severe malaria, as defined by the WHO [15], was included in this definition. Three clinical endpoints were used to evaluate the relationship between *Pf*-specific immune responses and malaria risk: 1) whether or not malaria was experienced, 2) the incidence of malaria, and 3) the time to the first malaria episode. Blood smears were prepared and venous blood samples collected during the two-week enrollment period (month 0), 14 days after the first episode of malaria (convalescence), and during a two-week period at the end of the six-month dry season (month 12). Hemoglobin was typed from venous blood samples. Stool and urine were examined at enrollment for the presence of helminth infections. Venous blood samples from ten healthy U.S. adult blood bank donors were analyzed as controls. Travel histories for these U.S. adults were not available, but prior exposure to *Pf* is unlikely.

### PBMC and plasma collection

Blood samples (8 ml for children and 16 ml for adults) were drawn by venipuncture into sodium citrate-containing cell preparation tubes (BD, Vacutainer CPT Tubes) and transported 20 km to the laboratory where they were processed within three hours of collection. Plasma and PBMCs were isolated according to the manufacturer's instructions. Plasma was stored at −80°C. PBMCs were frozen in fetal bovine serum (FBS) (Gibco, Grand Island, NY) containing 7.5% dimethyl sulfoxide (DMSO; Sigma-Aldrich, St. Louis, MO), kept at −80°C for 24 hours, and then stored at −196°C in liquid nitrogen. For each individual, PBMC and plasma samples from all time points were thawed and assayed simultaneously.

### Measurement of peripheral blood *Pf* parasitemia

Thick blood smears were stained with Giemsa and counted against 300 leukocytes. *Pf* densities were recorded as the number of asexual parasites/µl of whole blood, based on an average leukocyte count of 7500/µl. Each smear was evaluated separately by two expert microscopists blinded to the clinical status of study participants. Any discrepancies were resolved by a third expert microscopist.

### Hemoglobin typing

Hemoglobin was typed by high performance liquid chromatography (HPLC; D-10 instrument; Bio-Rad, Hercules, CA) as previously described [14].

### Stool and urine exam for helminth infection

At enrollment, duplicate stool samples were examined for *Schistosoma mansoni* eggs and other intestinal helminths using the semi-quantitative Kato-Katz method. To detect *Schistosoma haematobium* eggs, 10 ml of urine were poured over Whatman filter paper. One or two drops of ninhydrine were placed on the filter and left to air dry. After drying, the filter was dampened with tap water and helminths were eggs detected by microscopy.

### Geographic information system data collection

Latitude and longitude coordinates of study subjects' households were measured by a handheld global positioning system receiver (GeoXM; Trimble) and reported earlier [14].

### Antibody detection by ELISA

ELISAs were performed by a standardized method as described previously [70]. For both AMA1 and MSP1, a 1∶1 mixture of FVO and 3D7 AMA1 and MSP1 isotypes was used to coat the ELISA plates. The limit of detection for the AMA1 and MSP1 ELISA is based on the range of values that gives reproducible results at the Malaria Vaccine and Development Branch at NIAID where the assay is routinely performed. More specifically, the limit of detection is the ELISA unit value at the lowest point on the standard curve, multiplied by the dilution factor at which samples are tested. The minimal detection levels for the MSP1 and AMA1 ELISA assays were 11 and 33 ELISA units, respectively. For analysis, all data below the minimum detection level were assigned a value of one half the limit of detection (i.e. 6 units for MSP1, 17 units for AMA1). The limit of detection for the TT ELISA was not determined because we did not have access to TT-naïve serum.

### Memory B cell analysis

Antigen-specific MBCs were quantified by a modified version of the method developed by Crotty *et al* [71]. We found that adding IL-10 to the cocktail of polyclonal activators resulted in a six-fold increase in the efficiency of the assay (Weiss *et al*., unpublished observation). Briefly, PBMCs were thawed and cultured in 24 well plates at 37°C in a 5% CO_2_ atmosphere for six days in media alone (RPMI 1640 with L-Glutamine, Penicillin/Streptomycin 100 IU/ml, 10% heat-inactivated FBS, 50 µM β-Mercaptoethanol) or media plus a cocktail of polyclonal activators: 2.5 µg/ml of CpG oligonucleotide ODN-2006 (Eurofins MWG/Operon, Huntsville, AL), Protein A from Staphylococcus aureus Cowan (SAC) at a 1/10,000 dilution (Sigma-Aldrich, St. Louis, MO), pokeweed mitogen at a 1/100,000 dilution (Sigma-Aldrich), and IL-10 at 25 ng/ml (BD Biosciences). Cells were washed and distributed on 96-well ELISPOT plates (Millipore Multiscreen HTS IP Sterile plate 0.45 um, hydrophobic, high-protein binding) to detect antibody-secreting cells (ASCs). ELISPOT plates were prepared by coating with either: a 10 µg/ml solution of polyclonal goat antibodies specific for human IgG (Caltag) to detect all IgG-secreting cells; a 1% solution of bovine serum albumin (BSA) as a non-specific protein control; or 5 µg/ml solutions of either tetanus toxoid (TT), AMA1, or MSP1 to detect antigen-specific ASCs. For AMA1 and MSP1, a 1∶1 mixture of FVO and 3D7 isotypes was used to coat the ELISPOT plates. Plates were blocked by incubation with a solution of 1% BSA. For the detection of antigen-specific ASCs, cells were plated in duplicate in eight serial dilutions beginning with 5×10^5^ cells/well. For detection of total IgG ASCs cells were plated at six serial dilutions beginning at 4×10^4^ cells/well. After a five hour incubation of the cells in the ELISPOT plates, plates were washed four times each in PBS and PBS-Tween 20 0.05% (PBST), and incubated overnight with a 1∶1000 dilution of alkaline phosphatase-conjugated goat antibodies specific for human IgG (Zymed) in PBST/1% FCS. Plates were washed four times each in PBST, PBS, and ddH_2_O; developed using 100 µl/well BCIP/NBT for 10 minutes; washed thoroughly with ddH_2_O and dried in the dark. ELISPOTS were quantified using Cellular Technologies LTD plate-reader and results analyzed using Cellspot software. Results are reported as frequencies of MBCs per 10^6^ PBMCs after the six-day culture. The limit of detection of the MBC ELISPOT assay for this analysis was five ASCs per 10^6^ PBMC based on the average number of ASCs on the BSA control. Assay failure was defined as fewer than 1000 IgG^+^ ASCs per 10^6^ PBMCs after the six-day culture which resulted in the exclusion of 15% of individuals at month 0, 13.2% 14 days after the first malaria episode, and 7.3% at month 12. For individuals with a limited number of PBMCs, priority was given to performing the ELISPOT assay for MSP1, then TT, and then AMA1.

### Phenotypic analysis of B cell subsets

All phenotypic analyses were performed using mouse mAbs specific for human B cell markers conjugated to fluorophores as previously reported [18]. Fluorophore-conjugated mAbs specific for the following markers were used: PECy7-CD19, PE-CD20, APC-CD10, APC-CD27, PE-IgG (BD Biosciences, San Jose, CA) and FITC-CD21 (Beckman Coulter, Fullerton, CA). A four-color, two-stain strategy was used to identify B cell subsets as follows: plasma cells/blasts (CD19^+^ CD21^−^ CD20^−^), naive B cells (CD19^+^ CD27^−^CD10^−^), immature B cells (CD19^+^ CD10^+^), classical MBCs (CD19^+^ CD27^+^ CD21^+^), atypical MBCs (CD19^+^ CD21^−^ CD27^−^ CD10^−^) and activated MBCs (CD19^+^ CD21^−^ CD27^+^CD20^+^). FACS analyses were performed on a FACSCalibur flow cytometer (BD Biosciences) using FlowJo software (Tree Star, Ashland, OR).

### Statistical analysis

Data were analyzed using STATA (StataCorp LP, Release 10.0) and GraphPad Prism for Windows (GraphPad Software, version 5.01).The Kruskal-Wallis test was used to compare continuous variables between groups, and the Fisher's exact test was used to compare categorical variables. The Wilcoxon matched-pairs signed-rank test was used to compare measurements of the same parameter at two time points for the same individual. The correlation between different continuous measures was determined by using the Spearman correlation coefficient. The malaria-free probability over the twelve-month study period was estimated by the Kaplan-Meier curve, and the time to the first malaria episode was compared by the log rank test. Cox's proportional hazards model was used to assess the effect of the following factors on the hazard of malaria: age, gender, weight, ethnicity, distance lived from study clinic, self-reported bednet use, hemoglobin type, antigen-specific MBC frequencies and Ab levels. The same list of variables was included in logistic and Poisson regression models to determine their impact on the odds and incidence of malaria episodes, respectively. For all tests, two-tailed p values were considered significant if ≤0.05.

## Supporting Information

Figure S1 Correlative analysis of antibody levels and memory B cell frequencies specific for AMA1, MSP1, and tetanus toxoid. Shown are scatterplots of antibody levels versus memory B cell frequencies specific for (A) AMA1 (n = 64), (B) MSP1 (n = 67) and (C) tetanus toxoid (n = 128). Data are derived from venous blood samples drawn before the malaria season. Only individuals with both antibody and memory B cell data are included. For AMA1 and MSP1 the plots include individuals with antibody levels at or above the limit of detection of the ELISA. Individuals with ‘failed' ELISPOT assays are not included. As described in ‘Materials and Methods', assay failure was defined as fewer than 1000 IgG^+^ ASCs per 10^6^ PBMCs after the six-day culture. The Spearman's correlation coefficient is given for each plot.
(3.84 MB TIF)
