# Supplementary figures and images for: The Plasmodium falciparum-Specific Human Memory B Cell Compartment Expands Gradually with Repeated Malaria Infections
Source: PLoS Pathog. 2010 May 20;6(5):e1000912. doi: 10.1371/journal.ppat.1000912 (PMC2873912; doi:10.1371/journal.ppat.1000912)

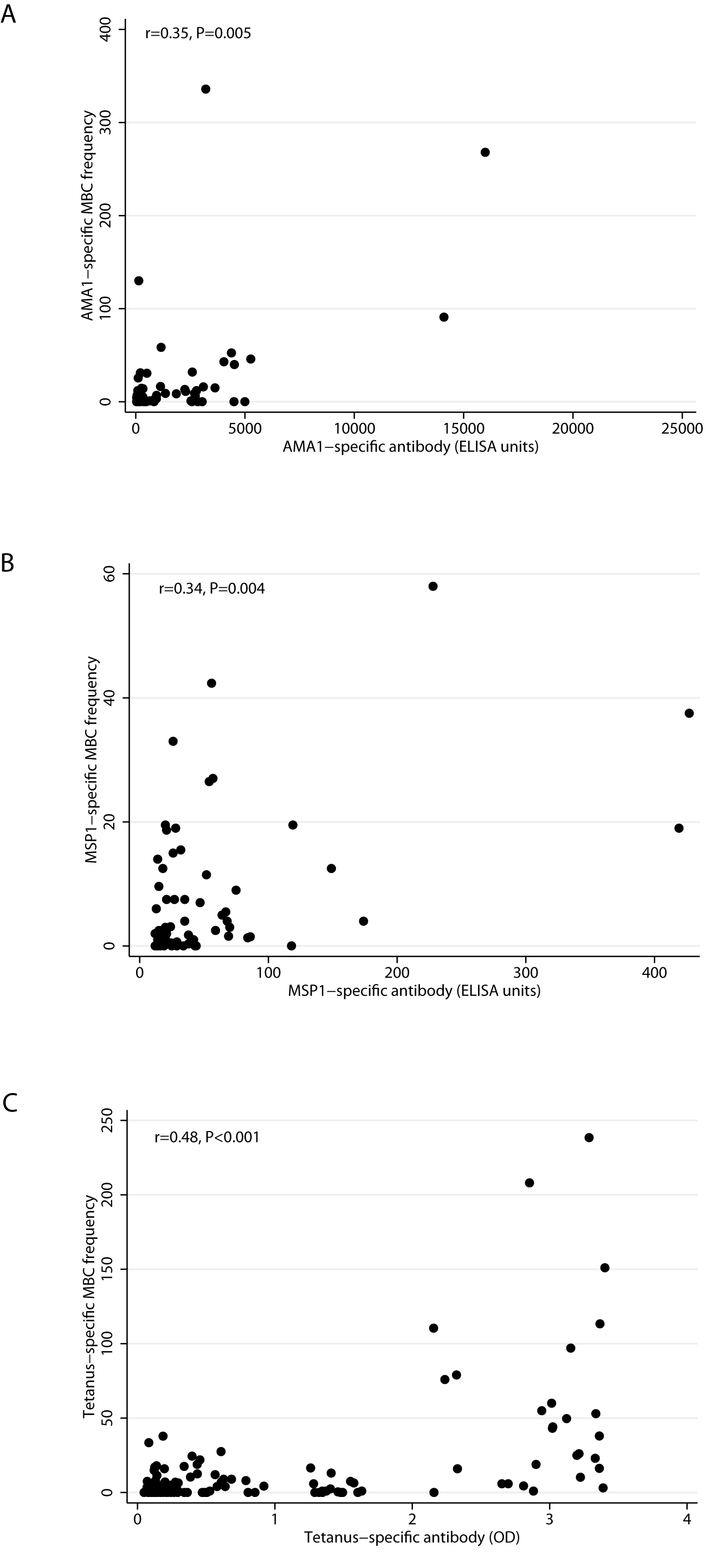

Supplement: Figure S1 — Correlative analysis of antibody levels and memory B cell frequencies specific for AMA1, MSP1, and tetanus toxoid. Shown are scatterplots of antibody levels versus memory B cell frequencies specific for (A) AMA1 (n = 64), (B) MSP1 (n = 67) and (C) tetanus toxoid (n = 128). Data are derived from venous blood samples drawn before the malaria season. Only individuals with both antibody and memory B cell data are included. For AMA1 and MSP1 the plots include individuals with antibody levels at or above the limit of detection of the ELISA. Individuals with ‘failed' ELISPOT assays are not included. As described in ‘Materials and Methods', assay failure was defined as fewer than 1000 IgG+ ASCs per 106 PBMCs after the six-day culture. The Spearman's correlation coefficient is given for each plot. (3.84 MB TIF) [file ppat.1000912.s001.tif]
